# Pressurised intraperitoneal aerosol chemotherapy (PIPAC): the first Australian experience

**DOI:** 10.1515/pp-2024-0028

**Published:** 2025-04-09

**Authors:** Katarina Foley, Jessica Reid, Suzanne Edwards, Timothy Price, Allan Zimet, Susan Woods, Markus Trochsler, Andrew Craig Lynch, Peter Hewett

**Affiliations:** Department of Surgery, 8703 The University of Adelaide, The Queen Elizabeth Hospital, Woodville South, SA, Australia; Adelaide Medical School, The University of Adelaide, Adelaide, SA, Australia; School of Public Health, The University of Adelaide, Adelaide, SA, Australia; 8703 Medical Oncology Service, The Queen Elizabeth Hospital, Woodville South, SA, Australia; Epworth HealthCare, Richmond, VIC, Australia; Precision Cancer Medicine Theme, South Australian Health and Medical Research Institute, Adelaide, SA, Australia; ANU School of Medicine and Psychology, The Australian National University, Canberra, ACT, Australia

**Keywords:** pressurised aerosolised intra-peritoneal chemotherapy, peritoneal carcinomatosis, peritoneal malignancy, peritoneal metastasis, peritoneal surface malignancies

## Abstract

**Objectives:**

Pressurised intraperitoneal aerosol chemotherapy (PIPAC) is a novel surgical technique for patients with peritoneal metastases not amenable to curative treatment. PIPAC delivers pressurised aerosolised chemotherapy using a hyperbaric capnoperitonem established laparoscopically. This study sought to investigate the feasibility and safety of PIPAC in an Australian population.

**Methods:**

We undertook a cohort analysis of prospectively-collected data on patients undergoing PIPAC across two Australian hospitals. Participants were planned to have three PIPAC procedures, each 6 weeks apart. Study outcomes included post-operative complications including 30-day mortality, length of stay (LOS) and patient quality of life (EORTC QLQ-C30 scores).

**Results:**

18 patients underwent 50 completed procedures. 13 patients had two or more PIPACs. The most common primary malignancy was colorectal cancer (n=8), followed by gastric cancer (n=4), appendiceal cancer (n=4) and mesothelioma (n=2). One grade four but no grade five complications occurred, with zero 30-day mortality. Median LOS was 1 day. Mean EORTC QLQ-C30 score increased from 47.8 at baseline to 53 post second PIPAC. Due to the heterogeneity of our cohort, survival analysis and statistical comparisons were unable to be made.

**Conclusion:**

PIPAC is feasible, safe and well tolerated in an Australian population with a lack of severe complications and zero 30 day mortality. Due to the small number of patients and the heterogeneity of our study’s sample, it was not possible to perform survival analysis. The study is nonetheless valuable as the first investigation of implementation of PIPAC in Australia.

## Introduction

Peritoneal surface malignancy involves tumour cells being deposited across the peritoneal surface lining the abdominal cavity and may be of either primary or secondary origin. Peritoneal metastasis (PM) is generally found in advanced cancers and is associated with poor prognosis and reduced QOL [1].

Palliative systemic chemotherapy is the mainstay of treatment except for select patients with resectable PM who can be considered for cytoreductive surgery (CRS) with or without heated intraperitoneal chemotherapy (HIPEC) [2]. A significant challenge when treating PM is the lack of responsiveness to systemic chemotherapy secondary to poor tissue penetration into peritoneal cancer cells due to the sparse peritoneal vascularisation and the plasma-peritoneal barrier [3]. Direct intra-abdominal administration of chemotherapy overcomes this limitation by increasing exposure of peritoneal cancer cells to chemotherapy, thus improving cytotoxicity [4].

Pressurised intraperitoneal aerosol chemotherapy (PIPAC) is a minimally-invasive surgical technique mostly reserved for patients with unresectable PM not suitable for CRS and HIPEC [5]. The aim of PIPAC is to prolong life and reduce symptomatic burden. Normothermic chemotherapy is delivered directly into the peritoneal cavity, using a hyperbaric capnoperitoneum established via laparoscopic ports [6], 7]. Chemotherapy is nebulised with a microinjector pump to create an aerosol, which is sprayed directly into the intra-peritoneal space [7]. Aerosolising chemotherapy facilitates more homogenous distribution within the peritoneal cavity [8]. Furthermore, the artificial pressure gradient established by the pneumoperitoneum overcomes tumoural interstitial fluid pressure, resulting in higher local drug concentration compared to conventional intra-peritoneal chemotherapy [8]. Bypassing the systemic circulation, PIPAC improves drug diffusion, diminishes systemic toxicity and reduces the required chemotherapy dose [8], 9]. Its minimally invasive nature spares the morbidity and mortality of a laparotomy such as with CRS and HIPEC.

Although trialled largely in Europe and Asia with promising results, PIPAC was not currently offered in Australia outside of this study. We sought to examine its feasibility across two Australian centres (The Queen Elizabeth Hospital (TQEH), South Australia and Epworth Health, Victoria) with a view to incorporating PIPAC into management for PM not suitable for curative resection, and to ensure equivalent results can be obtained in Australia.

## Methods

### Study design

This is a retrospective cohort analysis of prospectively collected data for patients commencing PIPAC across two Australian institutions between December 2019 and April 2022 with a follow-up period until November 2023. Eligible patients were those aged over 18 with peritoneal metastases who were not candidates for CRS and HIPEC. Additionally, participants had either (1) progression of their disease on standard palliative systemic chemotherapy, (2) refused systemic chemotherapy, or (3) were ineligible for further systemic chemotherapy due to treatment-related adverse events. Each case was discussed at a multi-disciplinary team meeting prior to referral for PIPAC. The study protocol aimed for participants to undergo at least three PIPAC procedures, each 6 weeks apart, and to be followed up for 1 year. Systemic chemotherapy was held at least 4 weeks prior to undergoing PIPAC to reduce the risk of cumulative chemotherapy toxicity [9].

### Ethics

For TQEH, Ethical Approval was granted on 15 April 2016 by the Central Adelaide Local Health Network (CALHN) Human Research Ethics Committee (HREC reference number: HREC/18/CALHN/383) with all participants providing written consent. Formal Ethical Approval was not required by Epworth Health, due to PIPAC having already been shown to be safe in previous international studies; hence, the local Institutional Review Board exempted the study from review as it was deemed to fall under audit and quality assurances. All data from both TQEH and Epworth Health have been managed appropriately under the Australian code of the Responsible Conduct of Research. The study also complied with the World Medical Association Declaration of Helsinki regarding ethical conduct of research involving human subjects.

### Operative procedure

All procedures were performed under general anaesthesia with the surgeon leading the operation having received specific PIPAC training. Laparoscopic access to the peritoneum was obtained via a midline 12 mm balloon port with a capnoperitoneum to 12  mmHg of CO [2] established. A 5 mm balloon port was inserted in the left upper quadrant or an alternative site if adhesions were present. The ports provided an air-tight seal to prevent leakage of vapour. Explorative laparoscopy ensued with any ascites present aspirated, measured and sent for cytology. The peritoneum and intra-abdominal structures were assessed for macroscopic disease and a PCI score calculated. Peritoneal biopsies were obtained from the right upper quadrant, right iliac fossa, left upper quadrant and left iliac fossa. All staff evacuated the operating theatre before chemotherapy was delivered via remote-control. Using a CapnoPen^®^ (Capnomed, Villingendorf, Germany) delivery system, chemotherapy was administered into the abdominal cavity at a constant flow rate of either 0.5 mL/s or 0.7 mL/s for up to 5 min, depending on volume. Chemotherapy agents included Oxaliplatin 92 mg/m^2^, Cisplatin 10.5 mg/m^2^ or Cisplatin 10.5 mg/m^2^ with Doxorubicin 2.1 mg/m^2^. The aerosolised chemotherapy was maintained in a steady state at a constant pressure for 30 min at 12  mmHg intraabdominal pressure. Following this, chemotherapy was evacuated using a high-efficiency particulate air (HEPA) filter closed aerosol waste system.

### Study outcomes

Data was extracted from medical records and entered into a secure electronic international database (REDCap). Additional information such as QOL questionnaire results, which could not be recorded in REDCap were stored in a password-protected Microsoft Excel™ document. Clinicopathologic data was collected for age, gender, primary malignancy, presence of metastases and prior cancer treatments, including surgery. PCI scores were recorded intra-operatively for each procedure as a quantitative assessment of macroscopic PM. Ascites volume was similarly recorded. Data was collected on post-operative complications, classified according to the Common Terminology Criteria for Adverse Events (CTCAE) version 4.0 [10]. LOS was recorded after each PIPAC. QOL was assessed only at TQEH prior to the first PIPAC and after each cycle using the European Organisation for Research and Treatment of Cancer Quality of Life Questionnaire (EORTC QLQ-C30) version 3.0 [11]. Data was expressed as either interquartile range or mean (±standard deviation)*.* Due to the small number of patients and the heterogeneity of our study’s sample, it was not possible to pool these patients in a survival analysis nor perform other statistical comparisons.

## Results

### Demographics and numbers

A total of 18 patients, 10 from TQEH and eight from Epworth Health, underwent 50 completed PIPAC procedures in addition to one non-access procedure and two procedures where there was insufficient intra-peritoneal space for PIPAC delivery.

The baseline clincopathologic characteristics are shown in Table 1. All those included had an Eastern Cooperative Oncology Group (ECOG) performance status of less than two. All had received systemic chemotherapy prior to participating in the study with half (n=9, 50 %) having received two lines of therapy. Treatment regimens for colorectal/appendiceal cancer included FOLFOX with Cetuximab followed by FOLFIRI and Avastin, Capecitabin/Oxaliplatin, dose reduced XELOX followed by XELIRI, FOLFOX, FOLFOXIRI, FOLFOX/XELOX, FOLFIRI, Capecitabin FOLFOX/FOLFIRI, FOLFOX/FOLFIRI/Cetuximab, Avastin, LONSURF/Avastin. Treatment regimens for the two participants with mesothelioma included Pemetrexed, and Cisplatin with Pemetrexed. Of the four participants with gastric cancer, chemotherapy regimens included FLOT (neo-adjuvant), FLOT (neoadjuvant) followed by FOLFOX with Nivolumab post op, intra-peritoneal Paclitaxel in combination with intravenous Cisplatin and Capecitabine, and FOLFOX with Herceptin. Out of the 18 participants, 12 (66.7 %) had undergone previous resection of their malignancy, and of those, eight had undergone bowel resection, two gastrectomy, one CRS and HIPEC and one total abdominal hysterectomy with bilateral salpingo-oophorectomy and appendicectomy. Although PIPAC is generally performed in patients with metastases confined to the peritoneum, three patients (16.7 %) were included who had low volume systemic metastases outside the peritoneum (Table 1).

**Table 1:** Participant characteristics.

Characteristics	Values, n (%) or median (Q1; Q3 interquartile range)
Primary malignancy	
Colorectal	8 (44.4)
Gastric	4 (22.2)
Appendiceal	4 (22.2)
Mesothelioma	2 (11.1)
Peritoneal metastases	
Synchronous	10 (55.6)
Metachronous	6 (33.3)
N/A	2 (11.1)
Gender	
Female	6 (33.3)
Male	12 (66.7)
History of prior systemic chemotherapy	
Yes	18 (100)
No	0 (0)
Previous surgical resection of malignancy	
Yes	12 (66.7)
No	6 (33.3)
Systemic metastases outside the peritoneum	
None	15 (83.3)
Lung	1 (5.6)
Hepatic	1 (5.6)
Retroperitoneal	1 (5.6)
Median number of PIPAC procedures	3 (1.25; 4)
Number of PIPAC procedures	
Total	50
1 only	5
2 only	2
≥3	13
Overall LOS post PIPAC; days	1 (1; 1)

**Figure 1:**
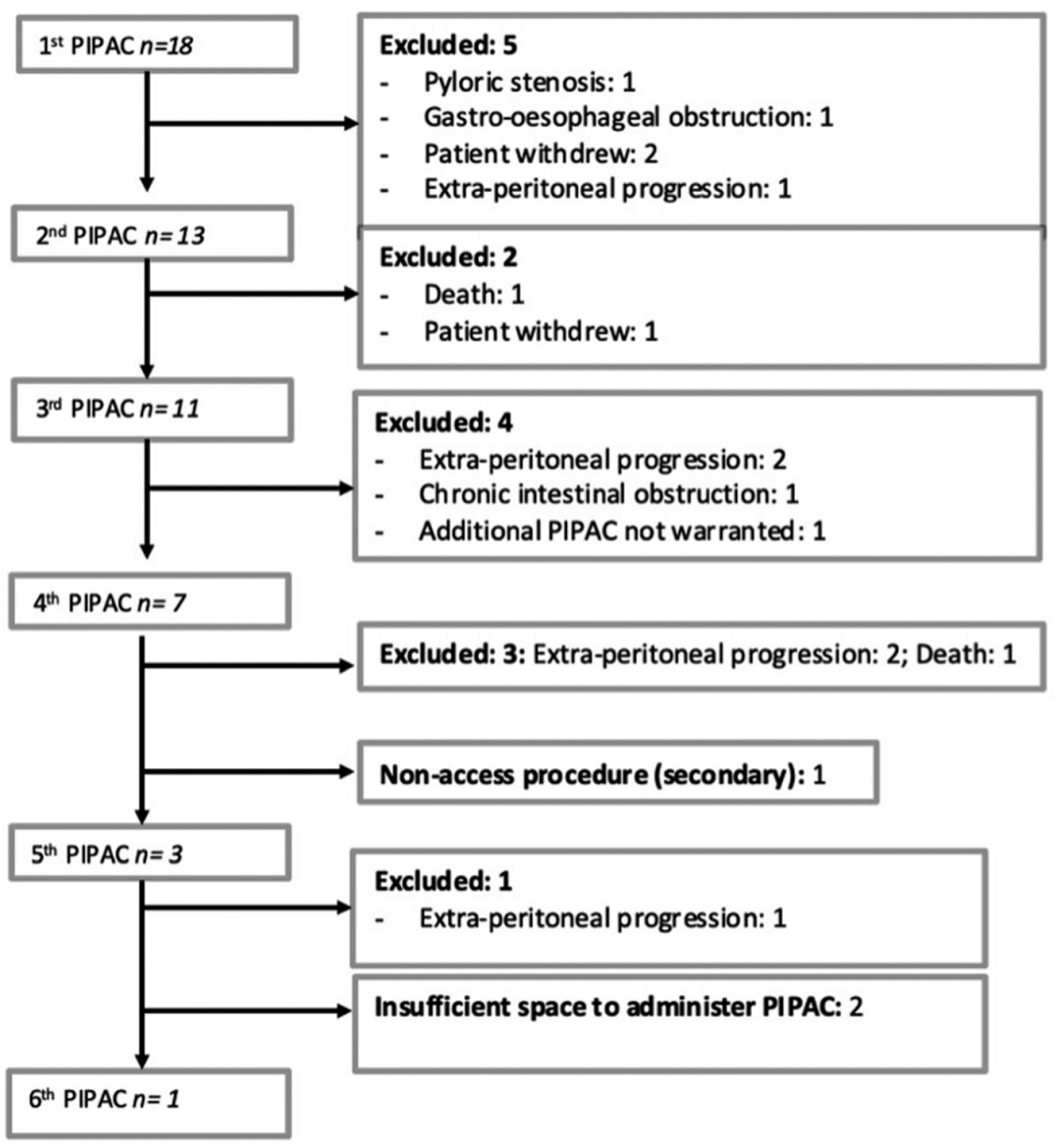
Flow chart of included patients.

One, two or three or more PIPAC procedures were performed for five (27.8 %), two (11.1 %), and 11 (61.1 %) participants, respectively. Progression of patients through the PIPAC study is detailed in Figure 1.

### Adverse events and length of stay

A total of 25 post-operative complications occurred, the most frequent being abdominal pain (n=9), followed by nausea and vomiting (n=6) (Table 2). Of the nine patients with significant post-operative pain, seven had received Oxaliplatin and two Cisplatin+/−Doxorubicin. Only one CTCAE grade four complication occurred (gastric perforation on entering abdomen requiring suture repair), most being either grade two (10) or three (13) complications and no grade five with a zero 30-day mortality rate. Our study had a total laparoscopic non-access rate of one, and included two patients with insufficient space for PIPAC delivery. Median post-operative LOS was 1 day (range 0.5–8 days).

**Table 2:** Post-operative complications.

Post-operative complications	Frequency
Abdominal pain	9
Nausea/vomiting	6
Small bowel obstruction	1
Diarrhoea	1
Constipation	1
Electrolyte abnormality (hypokalaemia)	1
Syncope/hypotension	1
Atelectasis	1
Ascites	2
Skin infection	1
Gastric perforation	1
Total	25

### Quality of life

Participant QOL data was only collected at one hospital (TQEH) using the EORTC QLQ-C30 questionnaire. Of these patients, nine out of 10 completed a baseline questionnaire, seven post first PIPAC, three post second PIPAC, two post third PIPAC and one post fourth PIPAC. The mean score was 47.8 at baseline, 50.7 post first PIPAC and 53 post second PIPAC.

### Response

Given the heterogenous nature of our study sample, we particularly report on the outcomes for the colorectal cancer group of patients (n=8) regarding PCI scores and ascites volume. Median PCI scores are detailed in Table 3. Of the eight colorectal cancer patients, seven underwent repeated PIPACs. Of these seven patients, PCI score decreased in three patients, increased in one patient and was unchanged in three patients. Median ascites volume at first PIPAC was 10 mL (range 0–6,900 mL), at second PIPAC 10 mL (range 0–4,500 mL) and at third PIPAC 50 mL (range 0–5,250 mL). Of the eight colorectal cancer patients, five had ascites at first PIPAC. Of these five patients, three had an overall decrease in the volume of ascites from first to last PIPAC and two had an overall increase.

**Table 3:** PCI scores.

	Median (Q1; Q3) Overall/colorectal
PCI at 1st PIPAC overall/colorectal only	28 (21; 32.8)/26.5 (21.3; 29.3)
PCI at 2nd PIPAC for all/colorectal only	26 (21; 32)/21 (19.5; 28)
PCI at 3rd PIPAC overall/colorectal only	26 (21; 31.5)/23.5 (21: 29)

## Discussion

Our results suggest PIPAC is a promising therapy for managing peritoneal surface malignancies not suitable for curative resection. We have demonstrated the safety and feasibility of PIPAC in an Australian population commensurate with international studies.

The minimally invasive and readily repeatable nature of PIPAC enables sequential procedures with the optimal effect occurring after three or more PIPAC cycles [5]. The majority of patients in our study (n=13, 72.2 %) underwent repeated procedures with a median number of three PIPACs; this finding corresponds with other studies and is indicative that repeated PIPACs are feasible for most patients [12], [13], [14], [15]. It could also be inferred that a median of three PIPAC cycles suggests our study appropriately selected participants, an important consideration as erroneous patient selection risks worsening QOL. Of the five patients who had only one PIPAC cycle, two patients did not proceed to a second PIPAC due to gastrointestinal tract obstruction. On reflection, these patients could perhaps have been identified earlier as not suitable for their initial PIPAC, by performing pre-operative imaging closer to their initial procedure.

Adhesions and peritoneal deposits associated with advanced PM can impede obtaining peritoneal access. Nonetheless, our study had a total laparoscopic non-access rate of one, in addition to two patients with insufficient space for PIPAC delivery (n=3), which is less than that of other studies reporting non-access rates of 15.4 % [16] to 17 % [17]. Consequently, the likelihood of obtaining safe access to the peritoneal cavity is an important consideration when determining suitability for PIPAC.

As our cohort of 18 patients comprised four different primaries (colorectal, gastric, appendiceal, mesothelioma), a limitation of our study was that we were unable to analyse survival or perform other statistical comparisons such as regression models to assess change in PCI score or ascites volume, given that outcomes post PIPAC, including survival, can differ markedly according to malignancy type [18]. However, as colorectal cancer was the most common primary, we chose to present simple statistics for PCI change and ascites volume for this group.

Similar to other studies such as Gockel et al. [19] and Rackauskas et al., [18] our results indicate that repeated PIPACs generally result in a reduction in ascites volume, although we were unable assess for statistical significance due to our heterogenous and relatively small sample size.

Regarding PM, whilst treatment modalities should aim to increase survival, it is vital that patient QOL is preserved. Nonetheless, QOL is often under-reported in studies on novel therapies in surgical oncology [20]. A limitation of our study is that QOL was assessed at only one of the two institutions (TQEH) with a diminishing number of returned EORTC QLQC-30 questionnaires with each PIPAC cycle, a tapering also noted by other studies [14]. Since our patient population was largely palliative, it is possible external factors contributed to poor compliance with returning completed questionnaires.

The single CTCAE grade four complication, no grade five complications, and a 30-day mortality of zero all support the safety of PIPAC; this aligns with other studies, which also report infrequent serious complications [17]. The most frequent complication was post-operative pain and, of the nine patients with significant post-operative pain, seven had received Oxaliplatin and two Cisplatin+/−Doxorubicin. This is in keeping with studies reporting PIPAC Oxaliplatin is associated with greater post-operative pain as compared with PIPAC Cisplatin+/−Doxorubicin [21].

Our median LOS across all PIPAC cycles remained at one day. While some studies had longer median LOS of three days [22], 23], our shorter stay could be accounted for by the fact that we did not routinely perform post-operative blood tests nor observe patients for several days post-op. The safety outcomes reported here are supportive of this approach in future trials and guidelines.

There is significant clinical heterogeneity in the majority of PIPAC studies, due to variation in the aetiology of PM [9], the different types of aerosolised chemotherapy utilised, or whether systemic chemotherapy was ceased during PIPAC treatment [24]. A systematic review conducted by Ploug et al. concluded that PIPAC studies need to be more standardised in their reporting to enable comparison of results [12].

Comparable to other PIPAC studies, a limitation is our small sample size, which risks underestimating the effect of PIPAC. Nonetheless, our number of 18 participants corresponds with other studies detailing the introduction of a PIPAC program in a single region, as illustrated by Rackauskas et al.’s first PIPAC program in the Baltic countries, which had 15 patients [18]. The fact that many PIPAC studies are retrospective and have a relatively small sample size hinders the ability to draw robust conclusions regarding efficacy. A potential solution is to form larger cohorts of patients from different centres and countries with standardised protocols for treatment and outcome measurement. Through publishing our results, we hope to increase awareness of this procedure, in particular in Australia, which may result in increased uptake and thus a larger series of patients. This could enable statistical analyses and assessment of the prognostic impact of repeated PIPAC cycles in an Australian population.

A potential future direction for PIPAC which is being investigated at our institution (TQEH) includes the addition of PIPAC to a curative treatment regimen for patients with gastric cancer to reduce the risk of peritoneal metastases occurring [25]. This is the first study of its kind to investigate PIPAC outside of the palliative setting. Another future direction is investigating the potential to tailor which chemotherapy is best administered during PIPAC for each patient’s malignancy, using patient-derived tumour organoids as a therapeutic response prediction platform, thus enabling personalised chemotherapy screening [26]. Although this has already been investigated for HIPEC [27], the use of patient-derived tumour organoids to individually tailor intra-peritoneal PIPAC chemotherapy has not been previously evaluated. We are continuing to assess the results of these future directions as more patients undergo PIPAC.

## Conclusions

In conclusion, the first experience of PIPAC in Australia demonstrates promising results regarding safety and feasibility with a lack of severe complications and zero 30-day mortality. Due to our relatively small and heterogenous sample size, our statistics were descriptive only as further statistical analyses must await larger series. This highlights the need for larger-scale randomised studies examining the efficacy of PIPAC. Our results are nonetheless valuable as they can be combined in meta-analyses with those from other countries, and may increase interest in implementing PIPAC in other Australian centres.
